# Animal Models of Alcohol Withdrawal

**Published:** 2000

**Authors:** Howard C. Becker

**Affiliations:** Howard C. Becker, Ph.D., is a professor in the departments of psychiatry and behavioral sciences and of physiology and neuroscience at the Medical University of South Carolina and is a research career scientist at the Department of Veterans Affairs Medical Center, Charleston, South Carolina

**Keywords:** animal model, chronic AOD (alcohol or other drug) withdrawal syndrome, in vitro study, in vivo study, locomotion, AODR (AOD related) seizure, convulsion, drug discrimination, dysphoria, autonomic nervous system, symptoms

## Abstract

One diagnostic criterion of alcohol dependence is the appearance of a withdrawal syndrome when alcohol consumption ceases. Researchers have used various animal models, including isolated brain cells, slices of brain tissue, and intact animals, to study the mechanisms and manifestations of withdrawal. Results from these experimental studies have demonstrated that many consequences of withdrawal found in animals resemble those observed in humans. Such signs and symptoms of alcohol withdrawal include enhanced activity of the autonomic nervous system; body posture and motor abnormalities; hyperexcitability of the central nervous system, including sensory hyperreactivity; convulsions; anxiety; and psychological discomfort. Researchers also have used animal models to study the electrophysiological correlates of withdrawal, as well as neurobiological mechanisms underlying alcohol dependence and withdrawal.

Excessive alcohol consumption over a prolonged period of time results in alcohol dependence, a maladaptive neurophysiological state that leads to a constellation of clinical signs and symptoms^1^ (i.e., alcohol withdrawal syndrome) when alcohol consumption is reduced drastically or stopped completely. These signs and symptoms typically reflect compensatory responses thought to represent the brain’s attempt to re-establish a functional balance (i.e., homeostasis) during continuous alcohol exposure (Becker 1999; Littleton 1998; Metten and Crabbe 1996). Consequently, these responses are the opposite of alcohol’s depressant effects on brain function. The clinical features of alcohol withdrawal generally fall into three categories: (1) hyperactivity of the autonomic nervous system, which regulates vital functions, such as heart rate, blood pressure, and respiration; (2) hyperexcitability of the central nervous system (CNS); and (3) distortions in sensation and perception (e.g., Anton and Becker 1995; Saitz 1998) (see table 1).

Each withdrawal sign and symptom usually emerges at a specific period following the reduction or cessation of alcohol consumption. In other words, withdrawal signs and symptoms follow a distinct temporal pattern. For example, tremors, anxiety, sleeplessness, restlessness, and nausea can begin as early as 6 hours after intoxication declines. Seizures usually occur within 48 hours after cessation of drinking, whereas delirium tremens (DTs) develop 1 to 4 days after the onset of acute alcohol withdrawal. The severity of withdrawal-related sequelae varies along a continuum, ranging from relatively mild symptoms after a single heavy drinking epsode (often referred to as a “hangover”) to more serious and complicated withdrawal symptoms resulting from chronic alcohol exposure. Overall, extensive variability exists among alcoholics with respect to the incidence and intensity of withdrawal signs and symptoms. This variability is undoubtedly related to a host of factors, such as amount, duration, and pattern of alcohol use; simultaneous abuse of drugs other than alcohol; compromised nutritional status; and coexisting illnesses (Saitz 1998). These factors, in turn, are influenced, to varying extents, by genetic forces.

Because of the diverse aspects of alcohol withdrawal and the countless intervening and confounding variables that may influence the syndrome’s manifestation, clinicians have difficulty in identifying risk factors, vulnerability, and underlying mechanisms of withdrawal in clinical studies of human alcoholics. The use of animal models to study alcohol dependence and withdrawal has enabled researchers to control both genetic and environmental factors contributing to alcohol withdrawal. Such studies have been critical in advancing knowledge about etiological factors and pathophysiological processes associated with alcohol withdrawal. This article reviews the experimental strategies used in animal models and describes the withdrawal signs and symptoms observed in such models.

## Experimental Strategies and Methods for Chronic Alcohol Exposure

Animal models have been extremely useful for investigating the factors that influence both risk and severity of alcohol withdrawal. These studies have employed a number of mammalian species, including chimpanzees and monkeys, dogs, cats, and rodents. Studies seeking to identify the biological, or intrinsic, influences (e.g., genetic predisposition, age, and gender) and environmental, or extrinsic, influences on alcohol dependence and withdrawal have primarily used rodents.

Two types of experimental strategies have demonstrated the importance of a genetic predisposition in shaping withdrawal symptoms. For the first strategy, those animals within one strain that show a particularly high or low propensity for alcohol withdrawal are selectively bred over several generations to generate two lines (i.e., selected lines) that differ only in their susceptibility to withdrawal, but not in the rest of their genetic makeup (i.e., genotype). The second approach involves comparing the susceptibility to withdrawal symptoms in numerous unrelated inbred animal strains with different genotypes. Other studies have identified additional biological variables that influence alcohol withdrawal. For example, experiments suggesting potential roles for certain hormones (e.g., steroid hormones that affect nerve cell function) have indicated that gender plays a role. Furthermore, investigations demonstrating developmental differences in neuroplasticity—that is, in the brain’s ability to adapt to changes in the environment—following chronic alcohol exposure have revealed the influence of age on susceptibility to withdrawal.

Researchers have evaluated the influence of environmental factors in animal studies in which investigators have systematically manipulated the animals’ alcohol exposure while otherwise controlling other environmental and biological influences. These studies have firmly established the modulating effects of alcohol dose (i.e., the amount consumed), duration of exposure, and pattern of exposure (i.e., the history of previous withdrawal experiences) on various parameters of the withdrawal syndrome. These findings have laid the foundation for using these models in expanding investigations on underlying mechanisms of withdrawal as well as for assessing the efficacy of various pharmacological treatment strategies for alcohol withdrawal.

To investigate the consequences of chronic alcohol exposure and withdrawal, researchers have conducted their studies both in the “test tube” (i.e., in vitro) and in intact animals (i.e., in vivo). Both of these approaches are discussed in the following paragraphs.

### In Vitro Models

With in vitro models, embryonic or neonatal nerve cells (i.e., neurons) extracted from various brain regions of rats or mice typically are grown and maintained in an artificial environment designed to simulate physiological conditions. To study the effects of alcohol exposure or withdrawal, alcohol is added to or removed from the fluid that supports and sustains the cells’ viability (i.e., the culture medium) (Hu and Ticku 1997). The advantage of these models is that the investigators can accurately control the dose and duration of alcohol exposure and determine alcohol’s direct effects on neuronal function under conditions that minimize extraneous influences. Such isolated systems also can be a disadvantage, however, because they preclude the analysis of potential complex interactions among different cells and brain regions that contribute to alcohol’s effects.

Alternatively, researchers have examined the effects of chronic alcohol exposure in thin slices of brain tissue that preserve the integrity of at least some of the cellular architecture and connections (i.e., synapses) of the tissue. In these studies, one can either treat the intact animal with alcohol and then extract and examine the brain slices or one can first extract the tissue slices and then treat them with alcohol once they are being cultured (Bailey et al. 1998; Thomas et al. 1998).

Collectively, these in vitro models have been used to study cellular and molecular events associated with chronic alcohol exposure and withdrawal. Although these techniques are well suited for mechanistic investigations (e.g., of the electrophysiological, biochemical, and molecular mechanisms contributing to withdrawal), the full spectrum of withdrawal symptoms and associated events (including behavioral and physiological measures) can best be studied in intact animals.

### In Vivo Models

A variety of in vivo models have been used to study the effects of chronic alcohol exposure and withdrawal. In these studies, alcohol administration is achieved through three main techniques, as follows:

- Because many animals do not voluntarily consume large (and consistent) alcohol amounts, alcohol administration often is forced—that is, imposed and controlled by the investigator. For example, the alcohol may be delivered directly to the stomach by intragastric infusion, or the animal may be continuously exposed to alcohol vapor in an inhalation chamber. Both of these strategies provide the experimenter with a great deal of control over critical variables of alcohol exposure, such as dose and timing (i.e., initiation, duration, and termination of exposure).
- For a less controlled approach, alcohol can be provided with the diet, most commonly as part of a nutritionally balanced liquid diet that constitutes the animals’ sole source of food and fluid. Under these conditions, animals typically consume sufficient quantities of alcohol to achieve dependence as evidenced by the emergence of withdrawal symptoms when the alcohol is omitted from the diet. With this approach, the investigator controls the duration of exposure, but the animal determines the dose and pattern of alcohol consumption.
- The third strategy relies on voluntary alcohol consumption. These models usually entail the use of animals that are genetically predisposed to high alcohol preference and drinking behavior when given a choice between alcohol and water.

With all of these strategies, researchers can achieve sustained alcohol exposure (i.e., persistent alcohol levels in the blood and brain) that results in the development of withdrawal signs and symptoms once alcohol administration is terminated. As with humans, withdrawal signs and symptoms in the animals wax as blood and brain alcohol levels drop, peak around the time when alcohol is completely eliminated from the body, and wane over the course of several days. Numerous variables influence the severity and timing of various components of the withdrawal syndrome in each animal, including the amount, duration, and pattern of alcohol exposure prior to withdrawal (Becker et al. 1997; Goldstein 1972; Macey et al. 1996; Watson and Little 1995).

Scientists also have developed several animal models to examine the consequences of repeated withdrawal experiences. In general, these studies have corroborated clinical findings obtained in humans indicating that people with a history of multiple detoxifications experience more severe and medically complicated withdrawal than do people experiencing their first withdrawal episode. This progressive intensification of withdrawal signs and symptoms may reflect a “kindling” or sensitization phenomenon. Traditionally, kindling refers to a process in which a weak electrical or chemical stimulus that initially causes no overt behavioral responses comes to result in behavioral effects (e.g., seizures) when administered repeatedly. The mechanisms underlying such a potential sensitization regarding withdrawal, however, remain unclear and are currently being investigated (Becker 1998, 1999; Becker and Littleton 1996).

## Withdrawal Signs and Symptoms in Animal Models

Numerous animal models involving diverse methods of alcohol exposure have identified a plethora of behavioral and physiological measures of withdrawal that to a great extent correspond to the withdrawal signs and symptoms observed in humans (see table 2) (for reviews, see Deitrich et al. 1996; Finn and Crabbe 1997; Friedman 1980; Metten and Crabbe 1996). The following sections review some of these findings.

### Enhanced Autonomic Activation

The autonomic nervous system has two parts. One part is the sympathetic nervous system, which accelerates the heart rate, constricts the blood vessels, and increases blood pressure. The second part is the parasympathetic nervous system, which slows the heart rate, increases the activity of the gastrointestinal (GI) tract and the glands, and relaxes certain muscles (i.e., sphincters) in the GI tract.

During alcohol withdrawal, the activity of the autonomic nervous system shifts to favor sympathetic activity (e.g., Hawley et al. 1994; King et al. 1994). As a result, laboratory animals undergoing withdrawal experience changes in cardiovascular functions (e.g., elevated heart rate and blood pressure) and GI functions (e.g., reduced food and water intake and diarrhea) (Crandall et al. 1989; Friedman 1980) similar to the effects observed in humans. Alcohol withdrawal, as well as alcohol exposure, also causes alterations in the animals’ ability to regulate body temperature. Finally, animals undergoing alcohol withdrawal exhibit tremors and piloerection (i.e., body hair standing up, which in humans results in “goose bumps”) (Friedman 1980; Frye et al. 1983).

### Body Posture and Motor Abnormalities

Rodents undergoing alcohol withdrawal demonstrate numerous indicators of impaired body function and motor activity. For example, in rats, tail stiffness is a characteristic feature of alcohol withdrawal. In addition, researchers have observed muscle spasms and abnormalities in body posture (i.e., rigidity) (see Friedman 1980) as well as alterations in gait and locomotor behavior ranging from depressed activity to wild fits of uncontrolled running. Furthermore, mice undergoing alcohol withdrawal exhibit various stereotypic behaviors, including backward walking and wall climbing (e.g., Becker et al. 1987). To measure these general withdrawal-related events, an investigator typically observes the animals during withdrawal and records abnormalities using a scoring schema. Because the assignment of scores using this approach is somewhat subjective, it is critical that the observer be unaware of the animals’ experimental histories (i.e., whether they were alcohol-treated or control animals) in order to minimize experimental bias.

### Sensory Hyperreactivity

Studies in human alcoholics have revealed a heightened reactivity to environmental stimuli during detoxification (e.g., Grillon et al. 1994; Krystal et al. 1997). To model this enhanced reactivity, researchers have exposed animals to various sensory stimuli. For example, studies measuring the animals’ startle response to auditory or tactile stimuli, such as an air puff, noted enhanced responsiveness during withdrawal (Macey et al. 1996; Pohorecky et al. 1986; Rassnick et al. 1992). Hyperreactivity during withdrawal also occurs in response to aversive stimuli, such as mild electroshocks. Finally, animals undergoing withdrawal commonly exhibit enhanced sensitivity to pain (i.e., hyperalgesia), for example, after the application of a heat stimulus to the tail or a paw (e.g., Gatch and Lal 1999).

### Convulsions

A hallmark feature of alcohol withdrawal is enhanced susceptibility to seizures (e.g., Becker 1999; Finn and Crabbe 1997; Victor 1970). Because it is relatively simple to identify, measure, and quantify the severity of seizures, this aspect of withdrawal has been studied extensively in animal models. These analyses have included both spontaneous and experimentally induced convulsions. To elicit seizures in animals undergoing withdrawal, researchers have used numerous procedures, as follows (for reviews, see Deitrich et al. 1996; Friedman 1980; Metten and Crabbe 1996):

- Exposure to a sensory stimulus, commonly a sound (i.e., an audio-genic stimulus)
- Handling the animal, for example, lifting it by the tail; with this approach, which is commonly used in mouse studies of alcohol withdrawal, the convulsions are scored depending on the degree of manipulation required to induce the response
- Exposure to mild electric stimuli, either to larger body areas (e.g., electroconvulsive stimulation) or to discrete brain regions (e.g., in the cortex or in subcortical brain areas)
- Administration of chemical substances (i.e., chemoconvulsants), either to the general bloodstream or directly to specific brain regions.

The convulsive behaviors elicited by these various experimental procedures during alcohol withdrawal appear to reflect a general state of heightened CNS excitability. (The electrographic correlates of this hyperexcitability are described in the sidebar, pp. 141–143.) For example, studies have found that in animals undergoing withdrawal, both the threshold amount (e.g., the current used for electrical stimuli or the dose of chemical stimuli) and the number of subthreshold stimulations required to elicit a convulsion were reduced compared with control animals (Deitrich et al. 1996; Friedman 1980; Metten and Crabbe 1996). Furthermore, in all cases the severity of the convulsive responses varied in a manner that presumably reflects the intensity of withdrawal-related CNS hyperexcitability. Interestingly, the timing for the expression of spontaneous and various experimentally induced convulsions during withdrawal differed, suggesting that different neural mechanisms underlie heightened CNS excitability at different times during the course of withdrawal (Gonzalez et al. 1989; Watson and Little 1995).

**Electrophysiological Indices of Withdrawal-Related CNS Hyperexcitability**

Hyperexcitability of the central nervous system (CNS) is an important feature of alcohol withdrawal. This hyperexcitability can be analyzed not only through behavioral measures, such as convulsions, but also through electrophysiological changes in brain activity. One approach to measuring electrophysiological changes is an electroencephalogram (EEG). An EEG records the combined activity of large ensembles of nerve cells (i.e., neurons) by tracing the electric potential (i.e., voltage) produced by those cells. EEGs show neuron activity as characteristic fluctuations in voltage (i.e., brain waves) that are detected by electrodes placed on the outside of the head, directly on the brain, or within the brain tissue. Characteristic changes in the brain waves, such as their size, amplitude, or frequency, may indicate certain psychological states, levels of consciousness, and neurological disorders.

Perturbations in neural activity cause either changes in spontaneous EEG activity or changes in response to the presentation of external stimuli, called evoked or event-related potentials (ERPs). Analyses of spontaneous EEG activity focus on changes in ongoing brain activity over time. Conversely, ERPs provide information about an individual’s ability to process and respond to sensory stimuli. For example, researchers can determine the time between the presentation of the stimulus and the appearance of the ERP. This is called the latency of the ERP. Computer-assisted analytic techniques also allow researchers to determine the frequencies of the predominant wave forms and to detect brain wave abnormalities that appear as greater-than-normal spikes and sharp waves (i.e., epileptiform activity)(see figure). Although the procedures for assessing spontaneous EEG activity and ERPs are distinct, the measures provide complementary information about the functional integrity of intact neural circuits.

EEG analyses have been useful in studying neurophysiological effects of acute and chronic alcohol exposure, which results in well-documented changes in spontaneous EEG activity, ERPs, and epileptiform activity in both humans and animal models (e.g., Porjesz and Begleiter 1996). During acute withdrawal after chronic alcohol consumption, alcoholics exhibit abnormalities in both spontaneous EEG activity and ERPs, as follows:

- Spontaneous EEG activity shows shifts in the predominant wave form frequencies and changes in the magnitude of the EEG signals within the different frequency bands.
- ERP alterations include increased amplitudes and decreased latencies.

Collectively, these neurophysiological changes suggest a rebound CNS hyperexcitability—that is, once alcohol with its depressant effects on brain activity is eliminated, the brain responds with greater-than-normal excitability (e.g., Biggins et al. 1995; Emmerson et al. 1987; Kaplan et al. 1985; Kathmann et al. 1996). Although some of these alterations in spontaneous EEG activity and ERPs dissipate with prolonged abstinence, other abnormalities persist (Porjesz and Begleiter 1996).

**Figure:**
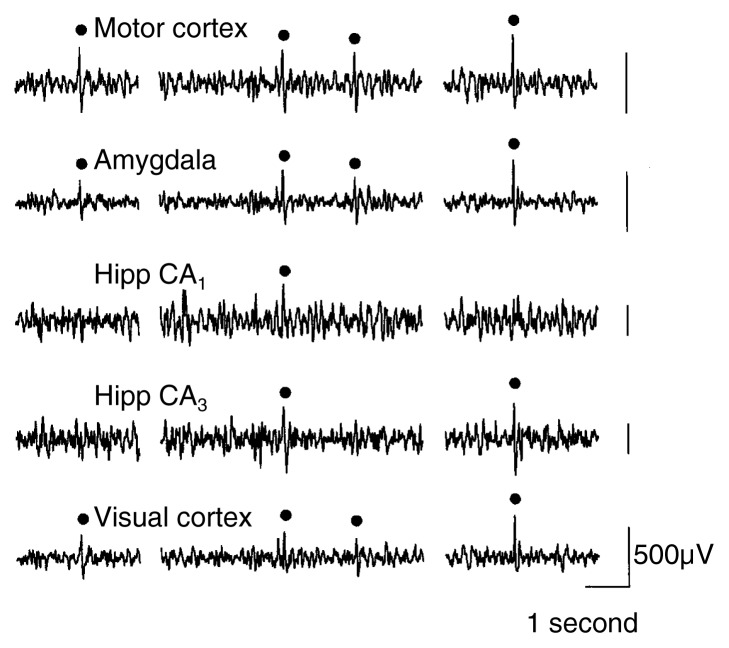
Sample electroencephalogram (EEG) recorded from a rat following alcohol withdrawal. Filled circles indicate computer-detected spikes and sharp waves, which are indicators of abnormal brain activity. Hipp = hippocampus. SOURCE: Adapted from Veatch and Gonzalez 1996.

Many of the EEG abnormalities also have been demonstrated in various animal models of alcohol withdrawal, including mice (Walker and Zornetzer 1974), rats (Ehlers and Chaplin 1991; Poldrugo and Snead 1984), cats (Perrin et al. 1975), and primates (Ehlers 1988). Indeed, studies using animal models have not only served to substantiate clinical findings, but have provided additional insight into subcortical epileptiform activity that may underlie and precede the convulsions associated with alcohol withdrawal. For example, the magnitude and frequency of epileptiform activity differs among various brain regions, with subcortical structures (especially hippocampus and amygdala^1^) exhibiting higher levels of spike activity than the cortex. Moreover, in rodent models, various regions of the hippocampus differ in their EEG response to chronic alcohol exposure and withdrawal. That is, the response of some hippocampal regions is influenced by the amount of alcohol exposure prior to withdrawal, whereas EEG abnormalities in other hippocampal regions are influenced more by the number of prior withdrawal episodes (Veatch and Gonzalez 1996). Finally, using various experimental approaches that involve in vivo and in vitro recording techniques, researchers are beginning to elucidate the biochemical mechanisms underlying these neurophysiological indices of withdrawal-related CNS hyperexcitability (e.g., Thomas et al. 1998; Whittington et al. 1995).

—Howard C. Becker

1Withdrawal signs constitute aspects of the syndrome that can be objectively measured (e.g., increased heart rate or blood pressure), whereas symptoms are of a more subjective nature and commonly reflect changes in mood or affect (e.g., irritability, anxiety, and depression).1Subcortical structures are brain areas located below the cortex, the thin layer of neurons (i.e., the “gray matter”) on the brain’s surface. Subcortical structures include the hippocampus, which plays a role in memory formation, and the amygdala, which is part of a group of brain structures that controls the expression of emotional behaviors.

ReferencesBigginsCAMackaySPooleNFeinGDelayed p3a in abstinent elderly male chronic alcoholicsAlcoholism: Clinical and Experimental Research1910321042199510.1111/j.1530-0277.1995.tb00985.x7485813EhlersCLERP responses to ethanol and diazepam administration in squirrel monkeysAlcohol53153201988322848310.1016/0741-8329(88)90072-9EhlersCRChaplinRIEEG and ERP response to chronic ethanol exposure in ratsPsychopharmacology10467741991188200610.1007/BF02244556EmmersonRYDustmanREShearerDEChamberlinHMEEG, visually evoked and event related potentials in young abstinent alcoholicsAlcohol42412481987362009110.1016/0741-8329(87)90018-8KathmannNSoykaMBickelREngelRRERP changes in alcoholics with and without alcohol psychosisBiological Psychiatry398738811996917270810.1016/0006-3223(95)00289-8KaplanRFGlueckBCHesselbrockMNReedHBCPower and coherence analysis of the EEG in hospitalized alcoholics and nonalcoholic controlsJournal of Studies on Alcohol461221271985399029710.15288/jsa.1985.46.122PerrinRGKalantHLivingstonKEElectroencephalographic signs of ethanol tolerance and physical dependence in the catElectroencephalography and Clinical Neurology39157162197510.1016/0013-4694(75)90005-x50212PoldrugoFSneadOCElectroencephalographic and behavioral correlates in rats during repeated ethanol withdrawal syndromesPsychopharmacology831401461984643146310.1007/BF00429722PorjeszBBegleiterHEffects of alcohol on electrophysiological activity of the brainBegleiterHKissinBThe Pharmacology of Alcohol and Alcohol DependenceNew YorkOxford University Press1996207247ThomasMPMonaghanDTMorrisettRAEvidence for a causative role of N-methyl-D-aspartate receptors in an in vitro model of alcohol withdrawal hyperexcitabilityJournal of Pharmacology and Experimental Therapeutics287879719989765326VeatchLMGonzalezLPRepeated ethanol withdrawal produces site-dependent increases in EEG spikingAlcoholism: Clinical and Experimental Research20262267199610.1111/j.1530-0277.1996.tb01638.x8730216WalkerDWZornetzerSAlcohol withdrawal in mice: Electroencephalographic and behavioral correlatesElectroencephalography and Clinical Neurology36233243197410.1016/0013-4694(74)90164-34130601WhittingtonMALambertJDCLittleHJIncreased NMDA receptor and calcium channel activity underlying ethanol withdrawal hyperexcitabilityAlcohol and Alcoholism3010511419957748267

Convulsive responses elicited during alcohol withdrawal are similar to those elicited during withdrawal from other CNS depressants (e.g., barbiturates and benzodiazepines). Furthermore, the convulsions associated with alcohol withdrawal may be exacerbated by drugs that promote convulsions and ameliorated by anticonvulsant drug treatment (e.g., Anton and Becker 1995). Consequently, these animal models of withdrawal-associated seizures have been pivotal in providing information about the conditions under which CNS hyperexcitability may be behaviorally expressed. Moreover, these models have offered insights into underlying mechanisms and effective treatment strategies for this potentially life-threatening aspect of alcohol withdrawal.

### Anxiety

Other prominent features of alcohol withdrawal in humans are anxiety and other symptoms of psychological discomfort. Scientists have used various experimental procedures to study behavioral measures of anxiety in animals during alcohol withdrawal (e.g., Emmett-Oglesby et al. 1990). For example, one can place rodents on an elevated “plus-maze,” a cross-shaped apparatus that is raised above the floor. Two arms of the cross are enclosed, and the others are open. When given a choice, rodents usually prefer to spend time on the enclosed arms. An increase or decrease in the time spent on the open arms is interpreted as a decrease or increase, respectively, in anxiety. Other procedures exploit the natural tendency for rodents to avoid novel and brightly illuminated open spaces. Studies found that alcohol initially produces an anxiety-reducing (i.e., anxiolytic) effect—that is, the animals spend more time on the open arms of an elevated plus maze or in bright, open spaces. Conversely, withdrawal from alcohol results in an anxiety-inducing (i.e., anxiogenic) effect, as indicated by more time spent on the enclosed arms of a plus maze and enhanced avoidance of bright, open spaces (e.g., File et al. 1992; Koob and Britton 1996).

Another model used to study withdrawal-related anxiety involves the drug discrimination paradigm. In this procedure animals are trained to discriminate between the subjective cues (i.e., “feelings”) associated with an anxiogenic compound (e.g., pentylenetetrazole [PTZ]) and an inactive compound (i.e., a placebo). The animals are shown two levers and receive a reward (i.e., are reinforced) for responding by pressing one lever following PTZ administration (i.e., when they are feeling “anxious”) and the other lever following placebo administration (i.e., when they are feeling “normal”). Discrimination learning is considered successful when the animals predominantly respond on the appropriate levers following PTZ or placebo administration. Animals undergoing alcohol withdrawal generally press the PTZ lever, even though they have not received the agent. This observation suggests that for the animals, the subjective experience of alcohol withdrawal is similar to that associated with PTZ administration (Gauvin et al. 1992; Lal et al. 1988). Agents that effectively alleviate anxiety during alcohol withdrawal can block this generalization—that is, animals receiving those agents no longer press the PTZ lever during withdrawal (Lal et al. 1988). Other studies have demonstrated that animals can clearly discriminate the subjective effects associated with alcohol intoxication (i.e., a “hangover”) and the subjective aspects of acute alcohol withdrawal (Gauvin et al. 1993).

The application of these various behavioral models of withdrawal-associated anxiety has played a key role in characterizing the anxiogenic component of the withdrawal syndrome, as well as in identifying conditions that maximize its expression. This work also has been instrumental in providing insight into underlying neurobiological mechanisms and into manipulations that may alleviate withdrawal-related anxiety.

### Psychological Discomfort

The most challenging aspect of studying alcohol withdrawal models in animals has been the associated psychological discomfort (e.g., distress and depression). Animals undergoing alcohol withdrawal have been noted to exhibit “irritability” and “aggressiveness”; however, these behaviors are subjective effects that are difficult to manipulate experimentally. One behavioral measure that has been postulated to reflect the stressful nature of withdrawal is the emission of sounds at ultrahigh frequencies (i.e., ultrasonic vocalizations) in rodents. These “distress” sounds increase dramatically in animals experiencing withdrawal, an effect that can be lessened by the administration of antianxiety drugs (Knapp et al. 1998).

One common characteristic of withdrawal-related discomfort is a reduced ability to derive pleasure from naturally rewarding events (i.e., anhedonia). Researchers have studied this subjective quality of alcohol withdrawal by assessing threshold levels for the perception of rewarding stimuli. In these experiments, animals are first trained to perform a task (e.g., press a lever) to receive a mild electric stimulus delivered to a site within the brain’s “reward center.” By systematically manipulating the stimulus intensity, the investigators can establish a threshold level of electrical stimulation that is perceived by the animal as being rewarding—that is, the current at which the animal responds for another stimulation 50 percent of the time. Such experiments found that alcohol initially lowers that reward threshold, because a weaker current is sufficient for the animal to perceive the stimulus as being rewarding. During alcohol withdrawal, however, the threshold for brain reward stimulation is significantly elevated (Schulteis et al. 1995). This model allows researchers to evaluate subtle aversive qualities of alcohol withdrawal that may contribute, at least in part, to the motivation for resuming drinking (i.e., relapse).

## Summary

Alcohol withdrawal syndrome comprises numerous signs and symptoms that emerge following cessation of drinking in people who excessively consume alcohol over a prolonged period of time. Many of these withdrawal signs and symptoms have been explored using both in vitro and in vivo animal models. Both of these research approaches have advantages and disadvantages, depending on whether one wants to study specific mechanisms underlying withdrawal or more general physiological and behavioral aspects of the syndrome. For example, in vitro models allow greater control over the experimental conditions and facilitate analyses of withdrawal-related processes at the cellular level. Alternatively, in vivo models enable researchers to investigate the effects of alcohol exposure and withdrawal on the entire organism. Such investigations facilitate analyses of behavioral and physiological consequences of withdrawal and allow researchers to evaluate the effectiveness of various treatment strategies.

Using a variety of model systems and alcohol administration protocols, researchers have been able to demonstrate many of the withdrawal signs and symptoms observed in humans. These studies have provided valuable insights into the etiology and underlying mechanisms of numerous withdrawal-related events, including enhanced autonomic nervous system activation, sensory hyperreactivity, convulsions, anxiety, and dysphoria. Based on these findings, researchers and clinicians hope to gain a better understanding of the sequelae of alcohol withdrawal, as well as develop novel and more effective treatment strategies for ameliorating those sequelae, thereby improving the chances of alcoholics to achieve abstinence.

## Figures and Tables

**Table 1:** Signs and Symptoms Associated With the Effects of Alcohol Withdrawal

Effects of Alcohol Withdrawal	Signs and Symptoms
Hyperactivity of the autonomic nervous system	Rapid heart beat (i.e., tachycardia)
	Elevated blood pressure
	Elevated respiration rate
	Profuse sweating
	Temperature dysregulation
	Nausea and vomiting
	Tremor
Hyperexcitability of the central nervous system	Agitation
	Anxiety
	Enhanced sensory reactivity
	Sleep disturbances
	Seizures
Sensory and/or perceptual distortions	Hallucinations
	Delirium, including confusion and disorientation (i.e., delirium tremens)

**Table 2:** Correspondence Between Alcohol Withdrawal Signs and Symptoms in Humans and Animals

Withdrawal Signs and Symptoms in Humans	Corresponding Withdrawal Signs in Animals
*Signs and symptoms as defined in the Clinical Institute Withdrawal Assessment for Alcohol, Revised (CIWA-Ar) questionnaire*	
Gastrointestinal disturbances (e.g., nausea and vomiting)	Reduced food and water intake; diarrhea
Tremor	Tremor
Autonomic hyperreactivity (e.g., excessive sweating)	Rapid heart beat (i.e., tachycardia); elevated blood pressure; central and behavioral thermal dysregulation; piloerection
Anxiety	Behavioral measures of anxiety (e.g., avoidance of bright, open spaces)
Agitation	Behavioral signs of irritability and aggressiveness
Sensory disturbances (i.e., tactile, auditory, and visual disturbances)	Sensory hyperreactivity (e.g., enhanced startle response)
Headache	N/A*
Disorientation and clouding of sensorium	N/A*
*Other clinical withdrawal symptoms used as diagnostic criteria as defined in the* Diagnostic and Statistical Manual of Mental Disorders,	
Fourth Edition	
Psychomotor agitation	Abnormalities in body posture, gait, and locomotor activity; stereotypic movements
Grand mal seizures	Electrographic and behavioral signs of central nervous system hyperexcitability
Insomnia	Abnormalities in the electroencephalogram

* N/A = No corresponding signs are available in animal models.
